# Treating ‘Septic’ With Enhanced Antibiotics and ‘Arthritis’ by Mitigation of Excessive Inflammation

**DOI:** 10.3389/fcimb.2022.897291

**Published:** 2022-06-09

**Authors:** Hyuk-Kwon Kwon, Christopher M. Dussik, Sang-Hun Kim, Themis R. Kyriakides, Irvin Oh, Francis Y. Lee

**Affiliations:** ^1^ Department of Orthopaedics and Rehabilitation, Yale School of Medicine, New Haven, CT, United States; ^2^ Section of Pulmonary, Critical Care and Sleep Medicine, Department of Internal Medicine, Yale School of Medicine, New Haven, CT, United States; ^3^ Department of Biomedical Engineering, Yale University, New Haven, CT, United States; ^4^ Department of Pathology, Yale School of Medicine, New Haven, CT, United States

**Keywords:** articular cartilage, chondroprotection, ERK, MRSA, septic arthritis, trametinib

## Abstract

Bacterial infection within the synovial joint, commonly known as septic arthritis, remains a clinical challenge as it presents two concurrent therapeutic goals of reducing bacterial burden and preservation of articular cartilage from destructive host inflammation. We hypothesized that mitigation of MRSA-induced inflammatory signaling could diminish destruction of articular cartilage in the setting of septic arthritis when used in conjunction with antibiotics. Herein, we provide evidence which supports a new therapeutic notion that concurrent antimicrobial therapy to address the ‘septic’ component of the disease with inflammation mitigation to manage the destructive ‘arthritis’ component. We established a murine model to mimic septic knee arthritis, as well as a variety of other inflammatory joint conditions. This murine septic arthritis model, in conjunction with *in vitro* and ex-vivo models, was utilized to characterize the inflammatory profile seen in active septic arthritis, as well as post-antibiotic treatment, *via* transcriptomic and histologic studies. Finally, we provided the clinical rationale for a novel therapeutic strategy combining enhanced antibiotic treatment with rifampin and adjuvant immunomodulation to inhibit post-infectious, excess chondrolysis and osteolysis. We identified that septic arthritis secondary to MRSA infection in our murine model led to increased articular cartilage damage compared to various types of inflammatory arthritis. The activation of the pERK1/2 signaling pathway, which is implicated with the mounting of an immune response and generation of inflammation, was increased in intracellular MRSA-infected synovial tissue and persisted despite antibiotic treatment. Trametinib, an inhibitor of ERK signaling through suppression of MEK1/2, alleviated the inflammation produced by the addition of intra-articular, heat-killed MRSA. Further, when combined with vancomycin and rifampin, mitigation of inflammation by pERK1/2 targeting improved outcomes for MRSA septic arthritis by conferring chondroprotection to articular cartilage and diminishing inflammatory osteolysis within bone. Our results support a new therapeutic notion that cell/biofilm-penetrating antibiotics alongside adjuvant mitigation of excessive intra-articular inflammation accomplish distinct therapeutic goals: reduction of bacterial burden and preservation of articular cartilage integrity.

## Introduction

Septic arthritis involves bacterial invasion into the joint space by either hematogenous spread or direct inoculation in the setting of trauma or surgery. It represents an orthopaedic and medical emergency given its capacity to inflict substantial, permanent damage to the articular cartilage in as little as 24 hours (Carpenter et al., 2011). The incidence of septic arthritis continues to increase due to variety of factors, including an aging global population, an increase in invasive orthopaedic procedures, and an increasing patient population on immunosuppressive regimens (Kaandorp et al., 1997; Mathews et al., 2010). *Staphylococcus aureus* (*S. aureus*) is the most common bacterial pathogen associated with septic arthritis. For methicillin-susceptible *S. aureus* (MSSA) joint infections, treatment typically incorporates urgent surgical irrigation followed by intravenous β-lactam antibiotics therapy, such as nafcillin and cefazolin (Bamberger and Boyd, 2005; Li et al., 2019). However, despite these interventions, septic arthritis still confers high morbidity and recurrence is not uncommon, especially in cases involving methicillin-resistant *S. aureus* (MRSA) (Weston et al., 1999; Dubost et al., 2002; Salgado et al., 2007). Worrisomely, despite antibiotic therapy and surgical drainage, patients with septic arthritis may suffer from disease sequelae such as an altered gait, diminished range of motion, limb length discrepancy, abnormalities of bone growth, recurrent septic arthritis, and progression to osteomyelitis, which may ultimately lead to fusion of a major joint or amputation of a limb (Vincent and Amirault, 1990; Wang et al., 2003). As such, more efficacious treatment regimens are crucial in ensuring optimal outcomes in patients burdened by MRSA septic arthritis.

A host inflammatory response against pathogens is an essential component in halting the progression of septic arthritis. However, excessive inflammation can mediate deleterious processes which may exacerbate structural damage to the joint secondary to the infection. This continued onslaught of inflammation can delay functional recovery due to excessive, painful swelling and cause further joint destruction and disease sequelae, even after the infection is controlled (Howard et al., 1976; Welkon et al., 1986). Thus, in addition to treating the inciting infection, the treatment of articular damage incurred by excess inflammation poses another challenge in the face of septic arthritis.

The inflammatory response mobilized by septic arthritis is largely mediated by the innate immune system. Recognition of pathogen-associated molecular patterns (PAMPs) and host-derived molecules released in the setting of tissue damage, termed damage-associated molecular patterns (DAMPs), both serve as crucial stimuli to combat infection (Zindel and Kubes, 2020; Gong et al., 2020). Receptors present within both the plasma membrane and cytoplasm of innate immune cells recognize PAMPs and DAMPs and mediate intracellular signaling pathways, such as those facilitated by mitogen-activated protein kinases (MAPKs) and nuclear factor kappa-light-chain-enhancer of activated B cells (NF-κB), to induce expression of immune response proteins and pro-inflammatory factors (Rahman and McFadden, 2011; Gong et al., 2020). MAPKs, classified as ERK, c-Jun N-terminal kinase (JNK), and p38, comprise a family of serine/threonine protein kinases that are implicit in the regulation of numerous intracellular signaling pathways including immune response, inflammation, proliferation, differentiation, and cell death (Thalhamer et al., 2008). Previous studies have reported that MAPK activation is increased within synovial fibroblast cells derived from rheumatoid arthritis patients compared to those derived from osteoarthritis patients, and induced by pro-inflammatory cytokines including tumor necrosis factor-alpha (TNF-α), interleukin-6 (IL-6), and interleukin-1 (IL-1) in human synovial cells (Schett et al., 2000). Previously, MAPK inhibitors have been shown to improve outcomes in a murine rheumatoid arthritis model through their anti-inflammatory effects, suggesting that MAPK inhibitors may be utilized to protect joints in the setting of other inflammatory processes, like septic arthritis (Thiel et al., 2007; Thalhamer et al., 2008; Singh et al., 2009). However, while some work on the impact of MAPKs on inflammatory arthritis has been presented, there are insufficient studies on the involvement of MAPKs in infectious processes and potential for MAPK inhibitors to serve as adjuvants to conventional antibiotic regimens in septic arthritis. In this study, we aimed to verify the function of MAPKs in septic arthritis, the role of MAPKs in mediating the inflammatory response incurred by MRSA infection, and the efficacy of combinatorial therapy involving antibiotics alongside MAPK inhibitors to control excessive inflammation mediated by PAMPs and DAMPs.

## Results

### Septic Arthritis Secondary to MRSA Infection Causes Severe Articular Cartilage Damage and Bone Destruction Distinct From Other Sources of Inflammatory Arthritis

**Figure 1:**
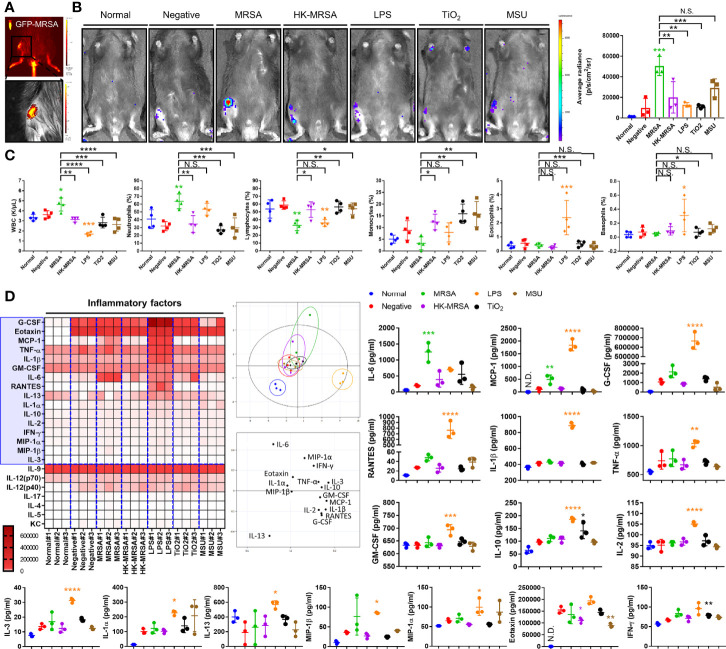
Variation in immunologic and inflammatory responses between inflammatory arthritis caused by infectious and non-infectious sources. **(A)** Successful intraarticular knee injection was verified by measuring the intensity of GFP immediately after administration of MRSA constitutively expressing GFP. **(B–D)** C57BL/6 mice were injected with MRSA (1×10^5^ CFU/10 μL), HK-MRSA (1×10^5^ CFU/10 μL), LPS (200 µg/mL), TiO_2_ (3 mg/mL), and MSU (200 µg/mL) into the knee joint space, with DPBS injection used as a negative control (*n* = 3-4 per group). **(B)** One day after injection, the generation of inflammation in the knee joint was assessed through measurement of MPO activity. **(C)** The total blood cell count and percentage of neutrophils, lymphocytes, monocytes, eosinophils, and basophils were measured using CBC analysis. **(D)** The generation of systemic inflammatory mediators, including cytokines, chemokines, and growth factors, was measured in serum and analyzed using principal component analysis; proteins that display significant variation in expression are highlighted within the blue box. Error bars show means ± SD. One-way ANOVA with Tukey’s *post hoc* analysis was used to assess statistical significance when compared to the negative control group or MRSA group (*p < 0.05 or **p < 0.01 or ***p < 0.001 or ****p < 0.0001; N.D., not detected and N.S., not significant).

To better characterize the chondro- and osteolytic patterns observed in both infectious and non-infectious arthritis, we constructed a murine model that may be utilized to mimic septic arthritis seeded through direct inoculation, as well as crystalline arthropathies (**Figure 1A**). We performed intra-articular knee joint injections of various factors. These included MRSA to induce septic arthritis, heat-killed MRSA (HK-MRSA) to observe the effects of inflammation mediated by PAMPs, lipopolysaccharides (LPS) to illustrate the effects of PAMPs found in gram negative septic arthritis, titanium dioxide (TiO_2_) as a common component of joint prostheses, and monosodium urate (MSU) to induce a gout-like crystalline arthropathy. MRSA infection significantly increased expression of myeloperoxidase (MPO) activity, a marker of increased inflammation secondary to neutrophilic reaction, more so than any other type of infectious or non-infectious inoculate (**Figure 1B**). Review of sera complete blood counts (CBC) showed that MRSA infection induced an increase in total white blood cells (WBC), similar to what is seen in the clinical setting (**Figure 1C**). In the model of MRSA septic arthritis and LPS inoculation, the percentage of neutrophils was increased whereas the percentage of lymphocytes decreased. The percentage of eosinophils increased only after LPS inoculation. Collectively, these CBC results suggest that systemic inflammation is generated after introduction of MRSA and LPS to the knee joint.

We further sought to characterize this inflammation through investigating markers of systemic inflammation, including cytokines, chemokines, and growth factors. In comparison to a negative control that simulated the intra-articular trauma of a joint injection, the production of systemic inflammation mediators, such as IL-6 and monocyte chemoattractant protein-1 (MCP-1/CCL2) were commonly increased in MRSA and LPS groups (**Figure 1D**). Other inflammatory cytokines, chemokines, and growth factors including granulocyte colony-stimulating factor (G-CSF), regulated on activation, normal T Cell expressed and secreted (RANTES/CCL5), IL-1-beta (IL-1β), TNF-α, granulocyte macrophage-colony stimulating factor (GM-CSF), IL-10, IL-2, IL-3, IL-1-alpha (IL-1α), IL-13, macrophage inflammatory proteins-1 alpha (MIP-1α/CCL3) were increased only in mice undergoing LPS inoculation.

**Figure 2:**
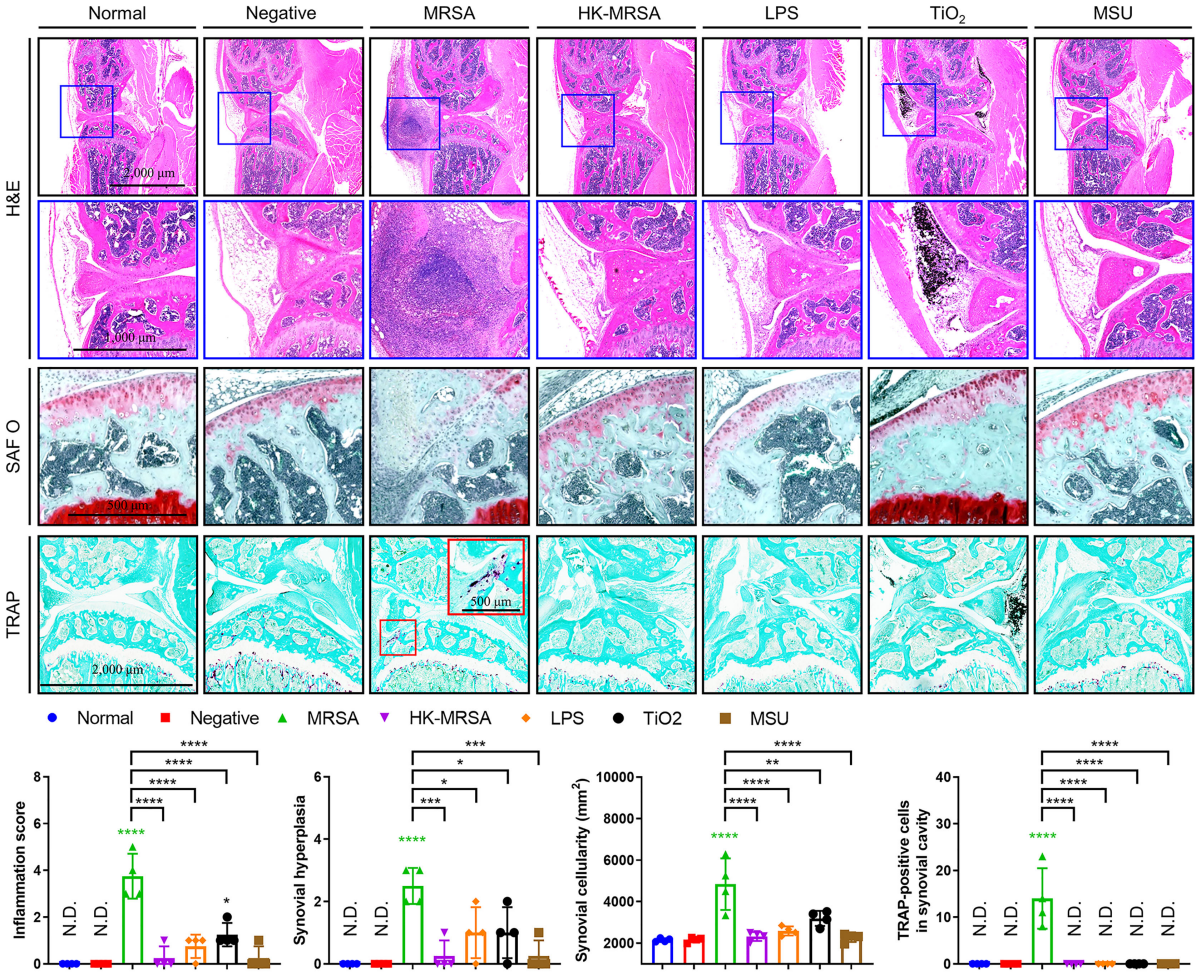
MRSA Septic arthritis induced inflammation, destruction of articular cartilage, and osteolysis. C57BL/6 mice received intraarticular knee injections of either MRSA (1×10^5^ CFU/10 μL), HK-MRSA (1×10^5^ CFU/10 μL), LPS (200 µg/mL), TiO_2_ (3 mg/mL), or MSU (200 µg/mL) with select mice receiving DPBS injection to serve as a negative control (*n* = 4 per group). Ten days after inoculation, mice were sacrificed, and tissues obtained for histological analyses. Paraffin-embedded knee joint tissues were sectioned and histologically stained with H&E, SAF O, or TRAP (scale bars: 2,000, 1,000, and 500 μm). The inflammation score, synovial hyperplasia, synovial cellularity, and TRAP-positive cells were measured using H&E- and TRAP-stained images. Error bars show means ± SD. One-way ANOVA with Tukey’s *post hoc* analysis was used to assess statistical significance when compared to the negative control group or MRSA group (*p < 0.05 or **p < 0.01 or ***p < 0.001 or ****p < 0.0001; N.D., not detected).

The results of the histopathological analysis showed that the inflammation score, synovial hyperplasia, synovial cellularity, and cartilage degradation were markedly increased in MRSA infection when compared to other types of infectious and non-infectious sources of inflammation (**Figure 2**). The introduction of TiO_2_ also increased inflammation score, synovial hyperplasia, and synovial cellularity, albeit lower than that seen with MRSA infection. Tartrate-resistant acid phosphatase (TRAP)-positive cells, which indicate activated osteoclasts, were significantly present at the resorption site of articular cartilage in mice with MRSA septic arthritis, but were absent in mice provided with other knee joint space inoculations. Taken together, mice in our MRSA septic arthritis model displayed infiltrating immune cells and inflammation that combined led to destruction of both cartilage and bone when compared to other groups of mice.

### Intraarticular MRSA Induced NF-κB and MAPKs Signaling Pathways Associated With Immune Responses, Inflammation, and Arthritis

**Figure 3:**
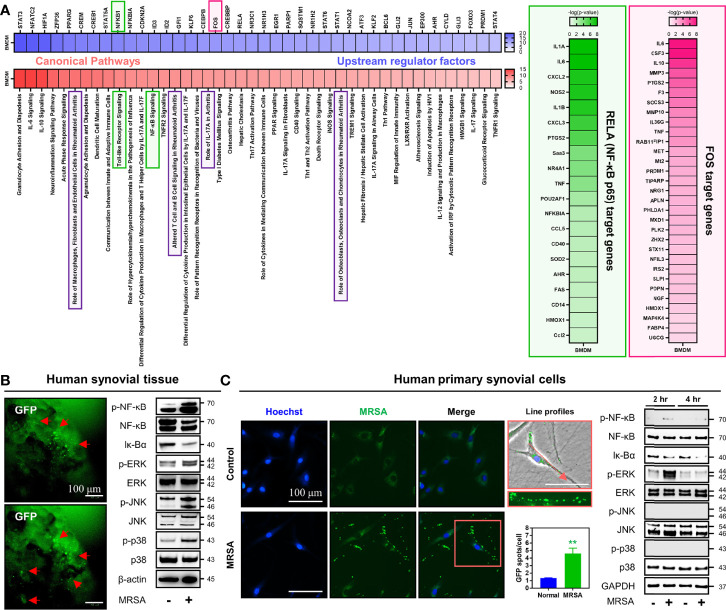
Infection with MRSA triggered NF-κB and MAPK signaling pathways associated with generation of immune responses, inflammation, and articular damage. **(A)** Transcriptome profiles were analyzed in primary BMDMs infected for four hours with MRSA using RNA-sequencing. We compared the normalized dataset with non-infected data, determined genes that were significantly increased in MRSA infection, and then analyzed canonical pathways alongside upstream regulating factors. The downstream genes regulated by NF-κB and FOS were verified and presented in the heatmap. **(B)** Human synovial tissue was infected with GFP-labeled MRSA (4×10^6^ CFU) for 24 hours and GFP expression was measured as a marker for ongoing infection (Scale bar: 100 μm). The expression of p-NF-κB, NF-κB, Iκ-Bα, p-ERK, ERK, p-JNK, JNK, p-p38, and p38 in whole protein extract obtained from MRSA-infected human synovial tissue was measured; β-actin was used as a loading control. **(C)** Human primary synovial cells were infected with GFP-labeled MRSA (4×10^6^ CFU) for 2 hours, at which time intracellular GFP expression was quantified (Scale bar: 100 μm). The expression of p-NF-κB, NF-κB, Iκ-Bα, p-ERK, ERK, p-JNK, JNK, p-p38, and p38 in whole protein extract derived from MRSA-infected synovial cells at two and four hours post-infection was measured; β-actin was used as a loading control. Error bars show means ± SD. Two-tailed unpaired *t*-test analysis was used to assess statistical significance when compared to the normal group (**p < 0.01).

To investigate the influence of MRSA septic arthritis on intracellular signaling pathways, we constructed and analyzed a data set obtained through RNA-sequencing of bone marrow-derived macrophages (BMDM) infected with MRSA. Signaling pathways related to numerous immune responses, inflammation, and those implicated in the development of arthritis were increased in MRSA-infected BMDMs (**Figure 3A**). Further, we identified upstream regulatory factors controlling genes whose expression were significantly increased in the presence of MRSA septic arthritis, which lead to an increase in transcription factors, such as NF-κB and fos proto-oncogene (FOS), that are related to immune responses, inflammation, and arthritis signaling pathways. Therefore, we hypothesized that the immune responses, inflammation, and arthritis secondary to MRSA infection receive upstream regulation through pathways featuring NF-κB and MAPK signaling.

We sought to investigate whether these findings could translate from our murine model into human-derived tissue using a combination of MRSA-infected human synovial tissue versus primary human synovial cells. In human synovial tissue infected with MRSA, activation of NF-κB was identified by increased expression of p-NF-κB with decreased expression of Iκ-Bα (**Figure 3B**). MAPK was also induced as evidenced by increased expression of p-ERK, p-JNK, and p-p38 (**Figure 3B**). MRSA-infected primary human synovial cells displayed numerous intracellular MRSA and were characterized by increased expression of p-NF-κB and p-ERK with decreased expression of Iκ-Bα (**Figure 3C**). However, the expression of p-p38 and p-JNK was unchanged when compared to controls. Taken together, we identified that MRSA infection increases the production of numerous pro-inflammatory molecules while mounting an immune response against infection. In turn, this may induce destruction of the joint mediated by NF-κB and ERK signaling pathways.

### Antibiotic Treatment Alone Was Effective in Reducing Bacterial Load, But Cartilage Destruction and ERK Activation Persisted Past Eradication of Infection

**Figure 4:**
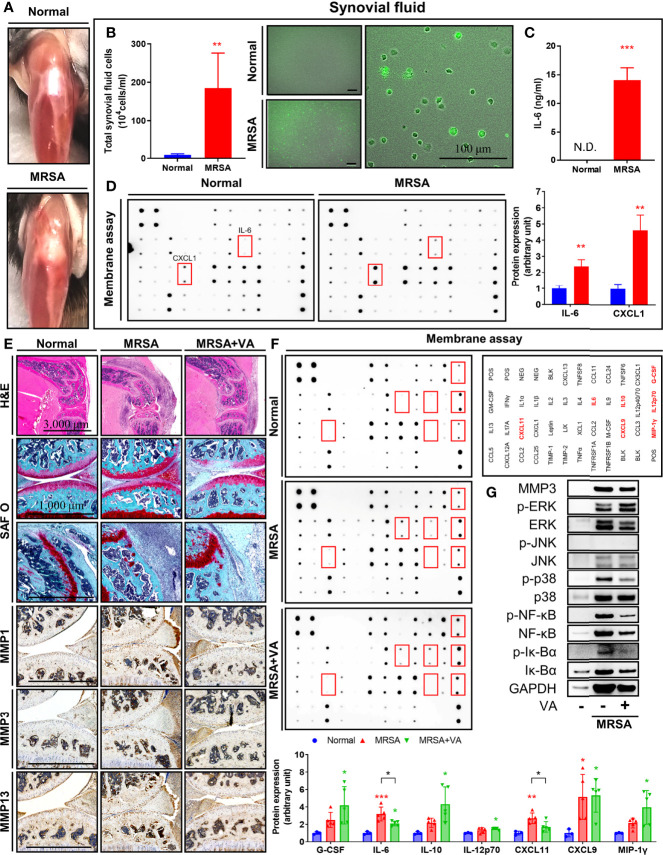
Intracellular MRSA persisted in synovial cells and ERK-mediated inflammation remained despite conventional vancomycin treatment. **(A–D)** C57BL/6 mice received intraarticular knee joint injections of MRSA (4×10^6^ CFU/10 μL), with analysis performed at one day post-infection (*n* = 3 per group). **(A)** Representative images of partially dissected knees. **(B, C)** Synovial fluid collected from knee joint in normal and MRSA infection. **(B)** Quantification of total synovial fluid cell count between normal and MRSA-infected mice (Scale bar: 100 μm). **(C)** The concentration of synovial IL-6 was quantified using ELISA. **(D)** Profiles of synovial pro-inflammatory factors were measured *via* membrane assays. **(E–G)** C57BL/6 mice were injected with MRSA (4×10^6^ CFU/10 L) into the knee joint. One day after infection, vancomycin (VA; 30 mg/kg) was subcutaneously injected for 3 days until eventually being sacrificed at 14 days post-inoculation (*n* = 3-4 per group). **(E)** Paraffin-embedded knee joint tissues were sectioned and that histologically stained with H&E and SAF O (Scale bar: 3,000 and 1,000 μm). Expression of MMP1, MMP3, and MMP13 was measured in paraffin-embedded knee joint tissues using immunohistochemistry assay. **(F)** Profiles of synovial pro-inflammatory factors were measured *via* membrane assays. **(G)** Expression of MMP3, p-NF-κB, NF-κB, p-Iκ-Bα, Iκ-Bα, p-ERK, ERK, p-JNK, JNK, p-p38, and p38 in whole protein extracted in synovial tissues were measured; GAPDH was used as a loading control. Error bars show means ± SD. One-way ANOVA with Tukey’s *post hoc* analysis or two-tailed unpaired *t*-test analysis were used to assess statistical significance when compared to the normal group or MRSA group (*p < 0.05 or **p < 0.01 or ***p < 0.001; N.D., not detected).

Knee joints infected with MRSA displayed signs of septic arthritis, including swelling and erythema at one day post-inoculation (**Figure 4A**). Synovial fluid aspirate revealed increased infiltrating immune cells, as well as numerous cells infected with intracellular MRSA (**Figure 4B**). Sharply elevated production of both IL-6 and keratinocytes-derived chemokine (KC/CXCL1) was evident within synovial fluid secondary to intraarticular MRSA infection, providing additional evidence for increased inflammation within the joint capsule (**Figures 4C, D**).

To mimic pharmacologic management guidelines (Liu et al., 2011), we initiated treatment of mice in our MRSA septic arthritis model with vancomycin. Upon sacrifice eleven days after completion of their vancomycin regimen, mice displayed signs of healing septic arthritis, including reduced erythema and edema, and histological analyses revealed decreased infiltrating immune cells (**Figure 4E**). The degradation of proteoglycans in articular cartilage and destruction of bone secondary to MRSA infection were inhibited after administration of vancomycin. Furthermore, immunohistochemistry revealed the expression of several matrix metalloproteinase (MMPs). Specifically, MMP1, MMP3, and MMP13, were induced by MRSA infection within synovial cells, immune cells, and chondrocytes (**Figure 4E**). While the administration of vancomycin suppressed expression of MMPs in the setting of MRSA infection, proteoglycan degradation and expression of MMPs within the joint space was still elevated compared to the non-infection group, supporting the need for novel adjuvant therapies to facilitate suppression of articular cartilage destruction. Production of IL-6 and C-X-C motif chemokine 11 (CXCL11) induced by MRSA infection was inhibited by vancomycin treatment, although the IL-6 level was still higher than normal group (**Figure 4F**). Moreover, when compared to the normal cohort, the production of G-CSF, interleukin-10 (IL-10), interleukin-12 (p70) (IL-12p70), monokine induced by gamma interferon (MIG/CXCL9), and macrophage inflammatory protein-1-gamma (MIP-1γ/CCL9) was increased in all settings featuring MRSA infection, regardless of treatment status. Finally, we identified that the expression of p-NF-κB and MAPK such as p-ERK and p-p38 in synovial tissue was increased in MRSA infection, but p-JNK expression was not detected (**Figure 4G**). Treatment of vancomycin reduced expression of p-NF-κB and p-p38, but expression of p-ERK remained despite pharmacologic management. Taken together, we identified that septic arthritis secondary to MRSA infection resulted in increased activation of ERK signaling which is associated with persistent cartilaginous damage despite antibiotic treatment. These results suggest that ERK may be implicated in articular cartilage destruction mediated by inflammatory processes and suggests a possible adjuvant treatment strategy to suppress residual inflammation.

### The ERK Inhibitor, Trametinib, Suppressed Pro-Inflammatory Markers Induced by HK-MRSA

**Figure 5:**
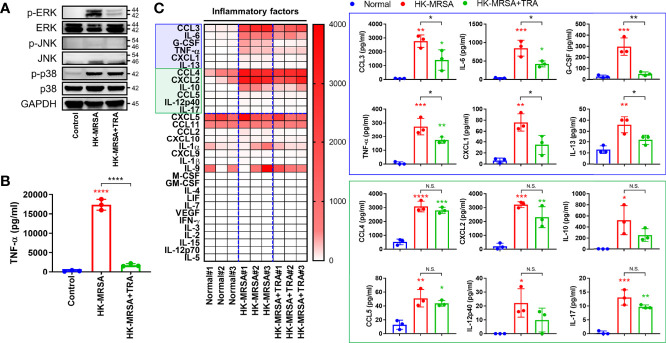
Trametinib inhibited production of pro-inflammatory markers induced by HK-MRSA. **(A)** The murine macrophage cell line, RAW264.7, was treated with trametinib (200 ng/mL) for one hour and inoculated with HK-MRSA (4×10^6^ CFU) for 30 minutes. Expression of p-ERK, ERK, p-JNK, JNK, p-p38, and p38 in whole protein was measured, with GAPDH serving as a loading control. **(B)** TNF-α concentrations in RAW264.7 cells under control conditions, inoculated with HK-MRSA (4×10^6^ CFU) for 12 hours, or treated with trametinib (200 ng/mL) for one hour and infected with HK-MRSA (4×10^6^ CFU) for 12 hours. **(C)** Murine blood treated with Trametinib (20 ng/mL) for 1 hour and then inoculated with HK-MRSA (4×10^6^ CFU) for 6 hours generated inflammation, with increased expression of cytokines, chemokines, and growth factors in serum (*n* = 3 per group). Blue and green boxes enclose inflammatory mediators that were significantly upregulated by HK-MRSA. Those encompassed by the blue box also represent inflammatory mediators with expression partially or completed rescued by trametinib treatment. Error bars show means ± SD. One-way ANOVA with Tukey’s *post hoc* analysis was used to assess statistical significance when compared to the control and normal group or HK-MRSA group (*p < 0.05 or **p < 0.01 or ***p < 0.001 or ****p < 0.0001; N.D., not detected). N.S., Not Significant.

Based on our previous results featuring *in vivo*, *ex vivo*, and *in vitro* models, we hypothesized that ERK activated by MRSA infection may play an important role in residual inflammation that continues to damage joints even after resolution of infection in cases of septic arthritis. To investigate the function of ERK in the generation of an intra-articular inflammatory response, we utilized the ERK inhibitor, trametinib. Trametinib is a pyridopyrimidine that is currently employed as treatment for metastatic melanoma through direct inhibition of mitogen-activated protein kinase kinase 1 and 2 (MEK1/2), an upstream signaling protein of ERK activation (Khan et al., 2020). We confirmed that murine macrophage cells (RAW264.7) inoculated with HK-MRSA displayed increased activation of both ERK and p38, as well as induced production of TNF-α (**Figures 5A, B**). Treatment with trametinib suppressed ERK activation in HK-MRSA-infected cells and, in turn, dramatically reduced production of TNF-α. Profiling of murine blood inoculated with HK-MRSA revealed that production of cytokines (e.g. IL-6, interleukin-13 (IL-13), and TNF-α), chemokines (e.g. macrophage inflammatory protein-1-alpha (MIP-1α/CCL3) and KC/CXCL1), and growth factors (e.g. G-CSF) were increased secondary to HK-MRSA (**Figure 5C**). Provision of Trametinib to blood samples partially rescued normal expression of a number of these cytokines, chemokines, and growth factors. However, select cytokines [IL-10, IL-12p40, and interleukin-17 (IL-17)] and chemokines [MIP-1-beta (MIP-1β/CCL4), CCL5, and macrophage inflammatory protein-2-alpha (MIP-2α/CXCL2)] remained elevated in the presence of HK-MRSA despite provision of trametinib. Taken together, we confirmed the anti-inflammatory effects of trametinib, suggesting that trametinib may be utilized to protect against articular cartilage destruction mediated by excess inflammation in the setting of septic arthritis.

### Adjuvant Treatment of ERK Inhibitor Protected Cartilage and Bone Destruction in Septic Arthritis

**Figure 6:**
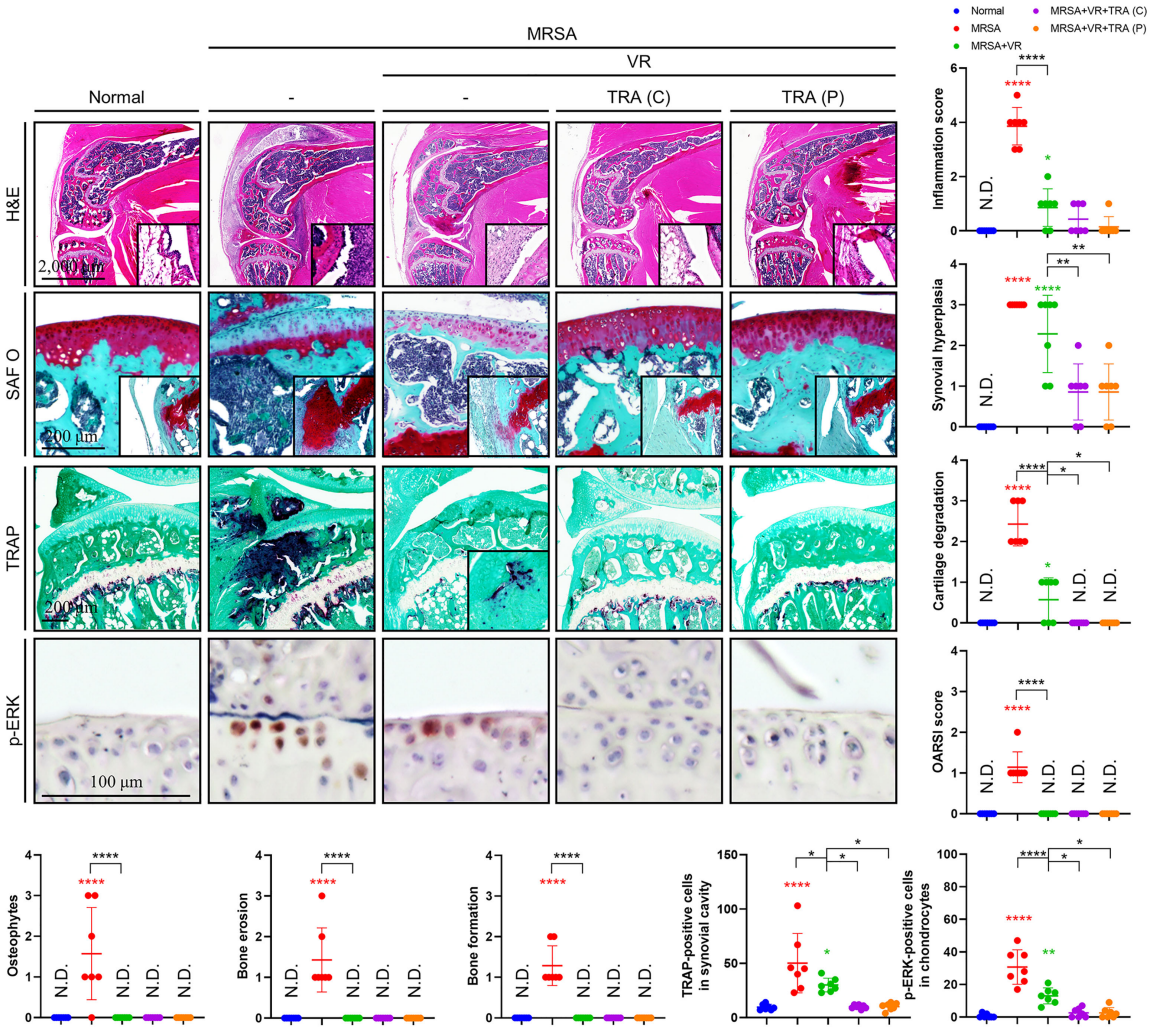
Adjuvant treatment of trametinib provided chondroprotection and inhibited inflammatory osteolysis. C57BL/6 mice received intraarticular knee joint injections of MRSA (1×10^6^ CFU/10 μL) (*n* = 7 per group). To test combination antibiotic treatment alone (abbreviated VR), vancomycin (30 mg/kg) and rifampin (20 mg/kg) were administered subcutaneously for 6 days after MRSA infection. To assess the viability of concurrent Trametinib supplementation [abbreviated TRA (C)], mice were given vancomycin (30 mg/kg) and rifampin (20 mg/kg) subcutaneously for 3 days and conjugated with a three-day trametinib regimen (1 mg/kg) beginning on post-injection day four. Finally, to test the utility of Trametinib at the end of antibiotic therapy [abbreviated TRA (P)], Vancomycin (30 mg/kg) and rifampin (20 mg/kg) were subcutaneously injected for 6 days after MRSA infection. On post-injection day seven, trametinib (1 mg/kg) was subcutaneously injected for three days. All models were sacrificed at 14 days post-MRSA injection. The inflammation score, synovial hyperplasia, cartilage degradation, OARSI score, osteophytes, bone erosion, bone formation, TRAP-positive cells, and p-ERK-positive cells were measured using H&E-, SAF O-, and TRAP-stained images (Scale bar: 2,000, 200, and 100 μm). Error bars show means ± SD. One-way ANOVA with Tukey’s *post hoc* analysis was used to assess statistical significance when compared to the normal group or MRSA group or MRSA+VR group (*p < 0.05 or **p < 0.01 or ***p < 0.001 or ****p < 0.0001; N.D., not detected).

Our previous results displayed that antibiotic treatment with vancomycin proved insufficient in resolving the post-infection inflammatory state in septic arthritis, leading to increased damage within the joint space despite eradication of infection. Moreover, we provided evidence to suggest that MRSA may sequester within the intracellular environment, and that trametinib inhibited inflammation incurred by the presence of MRSA. Therefore, we utilized a septic arthritis model to test the viability of a variety of combinatorial treatments featuring vancomycin alongside rifampin to facilitate destruction of intracellular MRSA with or without Trametinib. On histological review of our septic arthritis model, edema was observed along with infiltration of a large number of immune cells in the synovial tissues (**Figure 6**). Characteristics indicating destruction of articular cartilage and cancellous bone were present, specifically a large number of TRAP-positive osteoclasts and p-ERK-positive chondrocytes with severe articular cartilage damage in mice with untreated septic arthritis. In comparison, the combination of vancomycin and rifampin effectively alleviated inflammation and mitigated cartilage and bone destruction. Despite antibiotic therapy, elevated inflammation score, synovial hyperplasia, cartilage degradation, osteoclast numbers, and p-ERK-positive numbers remained relative to non-infected mice. However, inflammation, synovial hyperplasia, cartilage degradation, and osteoclasts number all improved in both models treated with trametinib in combination with antibiotics.

## Discussion

In this study, we present evidence that inflammatory factors may persist in MRSA-infected septic arthritis despite antibiotic treatment, which may exacerbate joint structural damage even after eradicating the underlying infection. We suggest that trametinib, an immune modulating drug, may be utilized to protect against further immune-mediated insults to cartilage and bone through inhibiting ERK-mediated inflammation in post-infectious MRSA septic arthritis.

We demonstrated that the inflammatory symptoms were exhibited in knees injected with MRSA, LPS, and TiO_2_. However, septic arthritis secondary to MRSA infection presented with the most severe knee joint destruction, with substantial damage to articular cartilage and bone compared to other types of infectious and non-infectious stimulators. These results support that MRSA septic arthritis induces stronger joint destruction compared to chronic inflammatory arthritis, which reinforces evidence that acute inflammatory arthritis requires more aggressive treatment. Persistent or recurrent septic arthritis and osteomyelitis may be attributable to the ability of *S. aureus* to penetrate eukaryotic host cells, thus limiting the efficacy of antibiotics such as vancomycin that primarily act in the extracellular space (Yu et al., 2020; Alder et al., 2020; Cahill et al., 2021; Kwon et al., 2021). Although vancomycin treatment was effective, its combination with rifampin was more effective, suggesting that the combination of effective intracellular and extracellular antibiotics could be more efficacious for severe septic arthritis. Moreover, we expect to be able to reduce recurrent infectious diseases including septic arthritis and osteomyelitis by more effectively eradicating intracellular bacteria.

In the synovial tissues of patients with rheumatoid arthritis, members of MAPKs are expressed, implicating an important role for MAPKs in the pathogenesis of inflammatory arthritis disease (Schett et al., 2000). Activation of MAPK members was increased in macrophages and fibroblasts in the synovial tissue of patients with rheumatoid arthritis, which coincided with increased IL-6, TNF-α, and IL-1 stimulation in synovial fibroblast cells (Schett et al., 2000). In the synovial tissue of human TNF-α transgenic mice that developed chronic inflammatory and destructive polyarthritis, the expression of p-ERK and p-p38 increased in macrophages along with the increased expression of p-ERK in fibroblasts, whereas p-JNK did not significantly change (Gortz et al., 2005). In this study, we identified increased expression of p-ERK and p-p38 in the MRSA-infected septic arthritis model and human synovial tissue along with intra-articular inflammation, while p-JNK was not significantly changed. Although conventional antibiotic treatment relieved the symptoms of septic arthritis, p-ERK expression remained elevated and coincided with inflammation and cartilage destruction, whereas the expression of other members of MAPKs were decreased. HK-MRSA, a non-viable MRSA, induced ERK activation along with inflammation, which was inhibited by trametinib, supporting that ERK is an important key factor for inflammation generation in non-viable MRSA mediated by antibiotics. Adjuvant treatment of trametinib along with antibiotics protected against inflammation, cartilage, and bone destruction in septic arthritis. Similarly, our study demonstrated that the expression of p-ERK and the production of inflammation were increased in an aggressive osteolysis model mediated by LPS, titanium, and breast cancer cells and that a potent inhibitor of the ERK treatment reduced inflammation and protected bone destruction (Seo et al., 2010; Lee et al., 2011; Back et al., 2021).

While these results are exciting, our work is not without limitations. Most notably, mice are not considered natural hosts of *S. aureus* (Schulz et al., 2017) and the immune response mounted by mice to intraarticular MRSA and the symptomatic improvement observed after provision of trametinib may not be conserved between murine models and human patients. Additional experimentation will be necessary determine whether these results are clinically translatable from species-to-species. Further, as septic arthritis is effectively a closed abscess, surgical drainage forms of cornerstone of management. We did not perform surgical drainage of septic joints within our murine models to minimize exposure to anesthesia and limit cohort mortality. If performed, this drainage could have improved outcomes in mice treated with vancomycin alone compared to other treatment groups. Finally, relatively large loading and maintenance doses of vancomycin are required in humans to reach therapeutic levels within the joint space. While we attempted to utilize a comparable treatment regimen to that used in the clinical setting [i.e., similar dosing by weight to current human guidelines (Rybak et al., 2020)], interspecies variation in drug metabolism and distribution may have led to a comparatively delayed antibiotic response and exacerbated joint space damage in our models.

Nonetheless, our results provide compelling evidence that the addition of cell-penetrating antibiotics alongside immunomodulation to conventional treatment strategies for septic arthritis may improve outcomes. These results may be readily translated from our murine model into the clinical setting. Given that patients with severe septic arthritis frequently develop pronounced damage to both bone and cartilage, trametinib may provide an exciting adjuvant therapy to further protect against chondro- and osteolysis caused by septic arthritis. Altogether, our work highlights the importance of controlling not only the infectious etiology of disease but also the deleterious influence of excessive inflammation in the setting of septic arthritis.

## Materials and Methods

### Cell Culture and Antibiotic Treatments

The RAW264.7 cell line was purchased from ATCC (San Diego, CA, USA) and cultured in high-glucose Dulbecco’s Modified Eagle’s Medium (DMEM; Thermo Fisher Scientific, Inc., Waltham, MA, USA) containing 1% of penicillin/streptomycin solution (Thermo Fisher Scientific, Inc.) and 10% of fetal bovine serum (FBS; Thermo Fisher Scientific, Inc.). Primary human synovial cells isolated from human synovial tissue (described below) were cultured in DMEM containing 10% of FBS and 1% of penicillin/streptomycin solution. All cells were incubated in a humidified atmosphere containing 5% CO_2_ at 37°C (Thermo Fisher Scientific, Inc.).

Vancomycin hydrochloride was purchased from Sigma-Aldrich Co. (St. Louis, MO, USA) Lipopolysaccharide (LPS) derived from *E. coli 0111:B4* and monosodium urate (MSU) were purchased from *In vivo*Gen (San Diego, CA, USA). TiO_2_ (<20 microns, 93%) was purchased from Alfa Aesar (Ward Hill, MA, USA). Rifampin was purchased from G-Biosciences (St. Louis, MO, USA). Trametinib was purchased from Selleck Chemicals (Houston, TX, USA).

### Fluorescent MRSA Culture and Preparation

The USA300-FPR3757 strain of MRSA expressive of green fluorescent protein (GFP) was provided by Alice Prince at Columbia University (Diep et al., 2006). Single MRSA colonies were planktonically cultured in lysogeny broth (LB; Invitrogen, Carlsbad, CA, USA) containing oxacillin (6 µg/mL; Sigma-Aldrich Co.) in a 35°C incubator for 24 hours. Heat-killed MRSA (HK-MRSA) expressing GFP were procured by incubating USA300-FPR3757 (1×10^7^ CFU or 4×10^8^ CFU/mL) at 65°CC for 2 hours.

### *In Vivo* Animal Experiments

All animal experiments were approved by the Yale University Institutional Animal Care and Use Committee (IACUC; Number: 2020-20129). Male C57BL/6J mice (10-12-weeks) were purchased from the Jackson Laboratory (Bar Harbor, Maine, USA). Using the knee injection method reported in a previous study (Kwon et al., 2021), MRSA (1×10^5^ CFU/10 μL), HK-MRSA (1×10^5^ CFU/10 μL), LPS (200 µg/mL), TiO_2_ (3 mg/mL), and MSU (200 µg/mL) were intraarticularly injected under the patella using a U-100 Micro-Fine IV Insulin Syringe (28-gauge needle; BD Biosciences, San Jose, CA, USA). Mice were sacrificed at 1 and 10 days post-injection for subsequent experiments. Alternatively, mice receiving MRSA (4×10^6^ CFU/10 μL) were intraarticularly injected under the patella using a U-100 Micro-Fine IV Insulin Syringe (28-gauge needle; BD Biosciences) and sacrificed at 1 day for subsequent experimentation.

For combination therapy testing, mice receiving systemic vancomycin treatment alone, vancomycin (30 mg/kg) was subcutaneously injected using a U-100 Micro-Fine IV Insulin Syringe (28-gauge; BD Biosciences) daily for three days after MRSA (4×10^6^ CFU/10 µL) infection of the knee joint. For mice receiving trametinib in conjunction with antibiotics, vancomycin (30 mg/kg) and rifampin (20 mg/kg) were subcutaneously injected for six days after induction of MRSA (4×10^6^ CFU/10 µL) infection within the knee joint. On day four of treatment, trametinib (1 mg/kg) was administered subcutaneously once daily in combination with the antibiotics for the following three days. Finally, to test the viability of trametinib after antibiotic treatment, vancomycin (30 mg/kg) and rifampin (20 mg/kg) were subcutaneously injected for 6 days after MRSA (4×10^6^ CFU/10 µL) infection of the knee joint. On post-injection day seven, trametinib (1 mg/kg) was subcutaneously injected for the next three days. All mice were sacrificed at 14 days and used for subsequent experiments.

### *Ex Vivo*: Human Synovial Tissue Experiments

Specimens of human synovial tissues were collected during elective total knee replacement surgery, which was approved by the Institutional Review Board (IRB; Number: 2000021232) of the PI’s institution. All patients signed an informed consent form for participation in the study and for the use of their biological tissues. Synovial tissue was transferred to a plate containing DMEM and then infected with MRSA (4×10^6^ CFU) for 24 hours. MRSA expressive of GFP in tissue was measured by the ZOE™ Fluorescent Cell Imager (Bio-Rad Laboratories, Hercules, CA, USA) and was used for subsequent experiments.

Primary human synovial cells isolation was achieved *via* incubation in collagenase type I (0.25%; STEMCELL Technologies, Vancouver, BC, Canada) for one hour. Afterward, remaining impurities were filtered through a 40 µm Nylon Cell Strainer (Corning Incorporated Life Science, NY, USA). These cells were cultured with complete DMEM and passages 3−6 of synovial cells were used for subsequent experiments. Primary human synovial cells (1×10^6^) were seeded on a 6-well plate (BD Biosciences) and grown overnight. These cells were infected with MRSA (4×10^6^ CFU) for two and four hours for subsequent experiments. At two hours, extracellular MRSA was removed by washing using DMEM and then stained with Hoechst 33342 (Thermo Fisher Scientific, Inc.) for 10 minutes. Fluorescence intensity was measured by BioTek CytationTM 5 Cell Imaging Multi-Mode Reader and analyzed using BioTek Gen5 software (BioTek Instruments Inc., Winooski, VT, USA).

### *Ex Vivo*: Murine Whole Blood Experiments

Fresh whole blood was collected from male C57BL/6J mice (10-weeks) *via* cardiac puncture using a 1 mL BD Slip-Tip Disposable Tuberculin Syringe (28-gauge; BD Biosciences) to transfer approximately 200 µL whole blood to a BD Vacutainer™ Plastic Blood Collection Tube with Lithium Heparin (75 USP Units; BD Biosciences). Murine blood was treated with trametinib (20 ng/mL) for 1 hour and then inoculated with HK-MRSA (4×10^6^ CFU) for 6 hours. Serum was isolated using centrifugation and used in the multiplex inflammation assay experiments described below.

### *In Vitro*: HK-MRSA Infection Experiments

RAW264.7 cells (2×10^6^) were seeded on a 6-well plate (BD Biosciences) and grown overnight in a humidified atmosphere containing 5% CO_2_ at 37°C. Cells were treated with trametinib (200 ng/mL) for 1 hour and then inoculated with HK-MRSA (4×10^6^ CFU) for 30 minutes and used in Western blot experimentation.

RAW264.7 cells (1×10^5^) were seeded on a 24-well plate (BD Biosciences) and grown overnight in a humidified atmosphere containing 5% CO_2_ at 37°C. Cell treated with trametinib (20 ng/mL) for 1 hour and then infected with HK-MRSA (4×10^6^ CFU) for 12 hours and used in the ELISA experiment.

### RNA-Sequencing Analysis

The construction of RNA-sequencing data was performed according to the reported experimental materials and protocols (Kwon et al., 2021). RNA-sequencing data are deposited in Sequence Read Archive (SRA) under the National Center for Biotechnology Information (accession number: PRJNA647064). We normalized significantly changed genes in MRSA-infected BMDM cells and analyzed canonical pathways related to genes with increased expression using by Ingenuity Pathway Analysis (Qiagen Bioinformatics, Hilden, Germany). Subsequently, we analyzed upstream regulators regulating genes with increased expression and identified genes regulated by NF-κB and FOS.

### Complete Blood Count Analysis

Whole blood was collected *via* cardiac puncture using a 1 mL BD Slip-Tip Disposable Tuberculin Syringe (28-gauge; BD Biosciences) and mixed with a 0.5 M EDTA pH 8.0 solution (1:10 ratio; AmericanBio, Natick, MA, USA). CBC analysis, including the number of white blood cells, and percentage of lymphocyte, monocyte, neutrophil, eosinophil, and basophil was measured using a Hemavet 950FS (Drew Scientific, Dallas, TX, USA) according to the manufacturer’s instructions.

### Myeloperoxidase Activity Analysis

To investigate MPO activity, murine models representing different types of infectious and non-infectious sources of intraarticular knee inflammation were intraperitoneally injected with XenoLight RediJect Chemiluminescent Inflammation Probe (Perkin Elmer, Santa Clara, CA, USA) according to the manufacturer’s instructions. Luminescence intensity was measured at an equal condition of excitation and emission using the IVIS^®^ Spectrum *In Vivo* Imaging System (Perkin Elmer) and then the region of interest at the knee joint was analyzed using the Living Image^®^ software.

### Synovial Fluid Analysis

10 µL of DPBS was intraarticularly injected into the knee joint and aspirated using a U-100 Micro-Fine IV Insulin Syringe (28-gauge needle; BD Biosciences). The aspirate was then transferred to a sterile tube. Synovial fluid cell number (1:10 dilution) was measured using a TC20™ Automated Cell Counter (Bio-Rad Laboratories) and the presence of GFP-labeled MRSA within synovial fluid cells was confirmed using the ZOE™ Fluorescent Cell Imager (Bio-Rad Laboratories).

### Enzyme-Linked Immunosorbent Assay (ELISA) Analysis

Levels of IL-6 in synovial fluid (1:100 dilution) were measured using a mouse IL-6 Uncoated ELISA Kit (Invitrogen) according to the manufacturer’s instructions. In comparison, levels of TNF-α in the supernatant medium were measured using a mouse TNF-α Uncoated ELISA Kit (Invitrogen) according to the manufacturer’s instructions. Relative concentrations of either IL-6 or TNF-α were measured with a BioTek CytationTM 5 Cell Imaging Multi-Mode Reader and analyzed using BioTek Gen5 software (BioTek Instruments Inc.).

### Inflammation Antibody Array Analysis

The production of inflammation-mediating cytokines, chemokines, and growth factors in synovial fluid was measured using the RayBio^®^ C‐Series Mouse Inflammation Antibody Array C1 (RayBiotech Inc., GA, USA) according to the manufacturer’s instructions. Briefly, the inflammation antibody array membranes were incubated with a blocking buffer for one hour. The membrane was incubated with 1 mL of sample buffer containing 1 µL of a synovial fluid sample overnight at 4°C. The following day, the membrane was incubated with Biotinylated Detection Antibody Cocktail for 90 minutes. The membrane was incubated with Streptavidin-Conjugated HRP for two hours. After finishing each step, the membrane was washed with Wash Buffer I thrice and then with Wash Buffer II thrice. The membrane was incubated with SuperSignal West Femto Maximum Sensitivity Substrate (Thermo Fisher Scientific, Inc.) and the resulting luminescence intensity was measured by ChemiDoc™ Touch Imaging System (Bio-Rad Laboratories). Luminescence intensity was analyzed by ImageJ software (Schneider et al., 2012) and normalized to positive control expression levels.

### Multiplex Cytokine, Chemokine, and Growth Factor Analysis

Serum samples, which were derived from *in vivo* and *ex vivo* models, were submitted to the Immune Monitoring Core Facility at Yale University and the number of inflammation-mediating cytokines, chemokines, and growth factors was measured using the Bio-Plex Pro Mouse Cytokine 23-plex Assay (Bio-Rad Laboratories) or MILLIPLEX MAP Mouse Cytokine/Chemokine Magnetic Bead Panel-Immunology Multiplex Assay from Millipore (Billerica, MA, USA) according to the manufacturer’s instructions. Principal component analysis (PCA) was performed with QStudioMetrics (https://github.com/gmrandazzo/QStudioMetrics).

### Histological Evaluation

Knee joint tissue was fixed with PROTOCOL™ 10% Buffered Formalin (Thermo Fisher Scientific, Inc.) and decalcified with 10% EDTA solution (Sigma-Aldrich Co.) for 3 weeks at 4°C or Immunocal™ Decalcifier (StatLab, McKinney, TX, USA) for one day at 4°C. Paraffin-embedded tissue sections were stained with hematoxylin and eosin (H&E), safranin O (SAF O), and tartrate-resistant acid phosphatase (TRAP) at the Orthopaedic Histology Core Facility of Yale University. Images of the stained knee joint tissues were captured with a BioTek Cytation 5 Cell Imaging Multi-Mode Reader (Bio-Tek Instruments Inc.). The inflammation score, synovial hyperplasia, cartilage degradation, OARSI scores, osteophytes, bone erosion, and bone formation were measured according to established guidelines (Kwon et al., 2021). Synovial cellularity was counted by QuPath software using H&E-stained images (Bankhead et al., 2017). Osteoclast numbers and p-ERK-positive cells were directly counted by TRAP-, and p-ERK-stained images.

### Immunohistochemistry (IHC) Analysis

Experimentation involving IHC was performed according to the well-established experimental materials and protocols (Kwon et al., 2021) featuring incubations with primary antibodies, including MMP1 (Abcam, Cambridge, MA, USA, cat. number ab137332), MMP3 (Abcam, cat. number ab52915), MMP13 (Abcam, cat. number ab39012), and p-ERK (Cell Signaling Technology Inc., Danvers, MA, USA, cat. number #4370). Slides were photographed using a BioTek Cytation 5 Cell Imaging Multi-Mode Reader and then analyzed by BioTek Gen5 software (Bio-Tek Instruments Inc.).

### Protein Isolation and Western Blot Analysis

Extraction of whole protein from tissue was performed using Tissue Protein Extraction Reagent (Thermo Fisher Scientific, Inc.), while extraction of whole protein from cells was performed using Mammalian Protein Extraction Reagent (Thermo Fisher Scientific, Inc.). Protein concentrations were obtained using a bicinchoninic acid (BCA) protein assay kit (Thermo Fisher Scientific, Inc.) following manufacturer protocol and then prepared with 4x Laemmli protein sample buffer (Bio-Rad Laboratories) according to the manufacturer’s instructions. Western blot experiments were performed following established experimental materials and protocols (Kwon et al., 2021) using immunoblots with primary antibodies, including p-NF-κB (Cell Signaling Technology Inc., cat. number #3033), NF-κB (Cell signaling Technology Inc., cat. number #8242), p-Iκ-Bα (Cell signaling Technology Inc., cat. number #2859), Iκ-Bα (Cell signaling Technology Inc., cat. number #4814), p-ERK (Cell signaling Technology Inc., cat. number #4370), ERK (Cell signaling Technology Inc., cat. number #4695), p-JNK (Cell signaling Technology Inc., cat. number #9251 or Thermo Fisher Scientific, Inc. cat. number 44-682G), JNK (Cell signaling Technology Inc., cat. number #9252 or #9258), p-p38 (Cell signaling Technology Inc., cat. number #4511), p38 (Cell signaling Technology Inc., cat. number #8690), MMP3 (Abcam, cat. number ab52915), GAPDH (Cell signaling Technology Inc., cat. number #2118), and β-actin (Cell signaling Technology Inc., cat. number #3700).

### Statistical Analysis

All experimental data was analyzed using one-way analysis of variance (ANOVA) or two-tailed unpaired *t*-test in GraphPad Prism Version 8 (GraphPad Software Inc., La Jolla, CA, USA).

## Data Availability Statement

The datasets presented in this study can be found in online repositories. The names of the repository/repositories and accession number(s) can be found in the article/supplementary material.

## Ethics Statement

The animal study was reviewed and approved by Yale University Institutional Animal Care and Use Committee.

## Author Contributions

H-KK designed and performed experiments and analyzed the data. FL, TK, and IO conceived and designed the overall study as the Principal Investigator. CD and S-HK performed the experiments. All authors contributed to the writing and editing of the manuscript and approved the final manuscript.

## Funding

This research was supported by National Institutes of Health (NIH) National Institute of Arthritis and Musculoskeletal and Skin Diseases (NIAMS) grants AR056246 and AR068353.

## Conflict of Interest

The authors declare that the research was conducted in the absence of any commercial or financial relationships that could be construed as a potential conflict of interest.

## Publisher’s Note

All claims expressed in this article are solely those of the authors and do not necessarily represent those of their affiliated organizations, or those of the publisher, the editors and the reviewers. Any product that may be evaluated in this article, or claim that may be made by its manufacturer, is not guaranteed or endorsed by the publisher.
